# Hotspots of unimproved sources of drinking water in Ethiopia: mapping and spatial analysis of Ethiopia demographic and health survey Data 2016

**DOI:** 10.1186/s12889-020-08957-2

**Published:** 2020-06-08

**Authors:** Getahun Gebre Bogale

**Affiliations:** Department of Health Informatics, School of Public Health, College of Medicine and Health Sciences, Wollo University, P.O.B.1145 Dessie, Ethiopia

**Keywords:** Ethiopia, Hotspot, Spatial, Unimproved drinking water source

## Abstract

**Background:**

More than 35% of the Ethiopian population are using drinking water from unimproved sources. As per the United Nations’ Sustainable Development Goals, Ethiopia is aspiring to achieve universal and equitable access to safe and affordable drinking water for all by 2030. For these goals to be accomplished, it is important to map the country’s hotspot areas of unimproved source of drinking-water so that resource allocation and disease control can be optimized there. Therefore, the objective of this study is to map and identify hotspot areas of unimproved sources of drinking water in Ethiopia.

**Methods:**

A population based cross-sectional study was conducted in Ethiopia from January 18 to June 27, 2016. Data were collected from 10,064 households using a pretested and structured questionnaire. A stratified two-stage cluster sampling was employed where the enumeration areas were primary sampling units and households were secondary sampling units. Systematic sampling with probability proportional to size was employed to select samples. Datasets were cleaned and entered into SaTScan and ArcGIS software for mapping and analysis. The Global Moran’s I and spatial scan statistical tests (Bernoulli model) were done to explore the presence of clustering in the study area and local spatial clusters (hotspots) of unimproved sources of drinking water using ArcGIS version 10.3 and Kuldorff’s SaTScan version 9.4 software, respectively.

**Results:**

Unimproved sources of drinking water were spatially clustered in the study area (Moran’s *I*: 0.35, *p* < 0.05). A total of 143 significant clusters was identified. Of which, eight were most likely (primary) clusters and the other 135 were secondary clusters. The first spatial window which contains primary clusters was located in Amhara and Afar regions (LLR: 78.89, at *p* < 0.001). The other 33 spatial windows which contain secondary clusters were found in all regions, except Gambela region and Addis Abeba city administration (with a range of LLR: 10.09–78.89, *p* < 0.001).

**Conclusions:**

This study allowed the identification of important non-random clusters and hotspots of unimproved sources of drinking water. Therefore, these results will be determinant to help decision makers in their geographical interventions to combat problems related to drinking water quality.

## Background

An unimproved drinking water source refers to a source that by its nature it is not properly protected from outside contamination, in particular fecal contamination [1]. World Health Organization (WHO) and WHO/UNICEF joint monitoring program categorized sources of drinking water as improved and unimproved. Improved sources of drinking water include piped water into a dwelling, yard of plot, public tap or standpipe, tube-well or borehole, protected spring, protected dug well, and rainwater collection. On the other hand, unimproved sources of drinking water include unprotected well (dug); unprotected spring, cart with small tank or drum; tanker truck-provided water, surface water (river, dam, lake, pond, stream, canal, irrigation channel); and bottled water (because of potential limits on the quantity of water available to a household through this source, not the quality) [2–5]. Worldwide, contaminated water from unimproved sources is the biggest killer of children [6].

Worldwide, 785 million people lack basic needs of water for drinking service which includes 144 million people who are still reliant on surface water (unimproved water source). More than 2 billion people are using a drinking water from unimproved sources (contaminated with feces). Of this, the burden is more in sub-Saharan Africa countries. Consequently, people in these countries are vulnerable to water borne diseases. This contaminated drinking water is projected to cause 485,000 deaths due to diarrhea each year. By 2025, the estimation is that half of the world’s population will be deemed to live in water-stressed settings [7].

A study done in Benin revealed that more than 49% of the households surveyed used unimproved water sources for their daily use [8]. In this study area, the majority of water with very high risk (68.7%) was from unimproved sources of drinking water, particularly unguarded surface water and springs [5]. Surveys conducted at national level reported that 34–35% of households and 38.3% of population in the country are using drinking water from unimproved sources [5, 9]. A study done in Dabat district, Ethiopia, also reported that 51% of the respondents obtained their water from unimproved sources [10]. The time taken to collect water is greater for those using unimproved sources of water and for residents of rural areas, which is a double burden [5].

It is known that contaminated water can transmit diseases such as diarrhea, cholera, dysentery, typhoid, and polio, which are caused by the use of unsafe water from unimproved drinking water sources [7]. As expected, households who obtain their drinking water from improved sources are more satisfied with both water quality and availability than those who fetch it from unimproved sources [11]. Access to improved water sources significantly reduce waterborne diseases [12].

It is vital to have sustainable water sources to guarantee the human health. Sustainable Development Goal (SDG) target 6.1 appeals for universal and equitable access to safe and affordable drinking water. The target is tracked by the indicator “safely managed drinking water services” which means drinking water should be accessed from an improved water source that is found on the premises, available when needed, and free from any fecal and harmful chemical contaminants [7]. Ethiopia is planned to achieve this target by 2030 [5]. Even though improvement was shown at national level, the nation’s improved sources of drinking water are still at 65% in the household level (or 62% in the population level) which is too far apart from the target [9].

Studies done in Nepal and sub-Saharan countries revealed that there were substantial geographical disparities of sources of drinking water [12, 13]. Evidences on the spatial variation of unimproved sources of drinking water is still lacking at national level of geographical intervention. Optimized resource allocation and disease control may be maintained if these gaps are filled. For this reason, to achieve the aspiring target and fill the gaps in information, it is important to explore the spatial hotspots of unimproved sources of drinking water in Ethiopia and alert the decision makers about the specific administrative areas that are in greatest need of policy attention. It is, therefore, the aim of this study is to map and identify the spatial hotspots of unimproved sources of drinking water in Ethiopia.

## Methods

### Study setting

This study was conducted in Ethiopia having a total population of 112, 469,180 [14]. Ethiopia is located 3^o^-14^o^ N and 33^o^-48°E, at the eastern Horn of Africa. The country covers 1.1 million Sq. km and has a great geographical diversity, which ranges from 4550 m above sea level (mountain Ras-Dashen) down to the Afar depression to 110 m below sea level (Dalol). There are nine regional states and two city administrations subdivided into 68 zones, 817 districts and 16,253 *kebeles* (*lowest local administrative units of the country*) in the administrative structure of the country [9, 15].

### Study design, period and population

A population based cross-section design was employed to assess types of sources of drinking water among households from January 18 to June 27, 2016. The study populations were all Ethiopian people founded in the selected enumeration areas and households at the time of data collection [9, 15].

### Data source and measurement

Every 5 years, the Ethiopian Demographic and Health Survey (EDHS) has collected data on nationally representative samples of key indicators including household drinking water supply. A stratified two-stage cluster sampling was employed where the enumeration areas (EA) were primary sampling units and households were secondary sampling units. An EA is a geographic area covering on average 181 households. Data was collected by trained interviewers from 645 EAs (202 urban and 443 rural areas) using systematic random sampling proportional to size. A fixed number of 28 households per enumeration area were selected for households’ sample frame. A structured and pretested questionnaire included household related variables towards sources of drinking water. Sources of drinking water were categorized by their type by asking the respondents that “what is the main source of drinking water for members of your household?” Hotspot means a place where high values of clusters of unimproved sources of drinking water (UISDW) together.

The 2016 EDHS database used for this analysis was prepared in seven data structures; namely; Household (HR), People-all household members (PR), Women with complete interviews (IR), Men with complete interviews (MR), Couples (CR), Children < 5 of interviewed women (KR), and All births to interviewed women (BR) data files. Among them, the household data file (dataset) was used for this analysis.

For this analysis, according to WHO classification, source of drinking water was categorized as “unimproved” and “improved”. Location data were also taken from selected EAs. Both the survey and location datasets were accessed through the web page of International Demographic and Health Survey (DHS) Program [16]. After cleaning of the household dataset which does not have location data, 10,064 households/respondents (or 46,395 populations) from 621 EAs were included in this study.

### Data analysis

The Global Moran’s *I* and spatial scan statistical tests were done to explore the presence of clustering in the study area and local spatial clusters (hotspots) of unimproved sources of drinking water using ArcGIS version 10.3 [17] and Kuldorff’s SaTScan version 9.4 [18] softwares, respectively. Global Moran’s *I* statistic measures whether the patterns of unimproved sources of drinking water are dispersed, clustered or randomly distributed in the study area. Moran’s *I* values close to − 1 indicate unimproved sources of drinking water (UISDW) dispersed, whereas, Moran’s *I* close to + 1 and 0 indicate UISDW is clustered and distributed randomly, respectively. A statistically significant Moran’s *I* (*p* < 0.05) value leads to the rejection of the null hypothesis and indicates clustering of unimproved sources of drinking water [15, 19].

The spatial scan statistic uses a scanning window that moves across the study area. Households using unimproved sources of drinking water were taken as “Cases” and households using improved sources of drinking water were taken as “Controls” to fit the Bernoulli model. The maximum spatial cluster size of < 50% of the population at risk with a circle radius of 100 km was used, as an upper limit, which allowed both small and large clusters to be detected and ignored clusters that contained more than the maximum limit. For each potential cluster, a likelihood ratio test statistic was used to determine if the number of observed cases within the cluster were significantly higher than expected or not. The primary and secondary clusters were identified and assigned *p-*values and ranked based on their likelihood ratio test, on the basis of 999 Monte Carlo replications [15, 20, 21].

## Results

### Spatial epidemiology and patterns of unimproved sources of drinking water

**Fig. 1:**
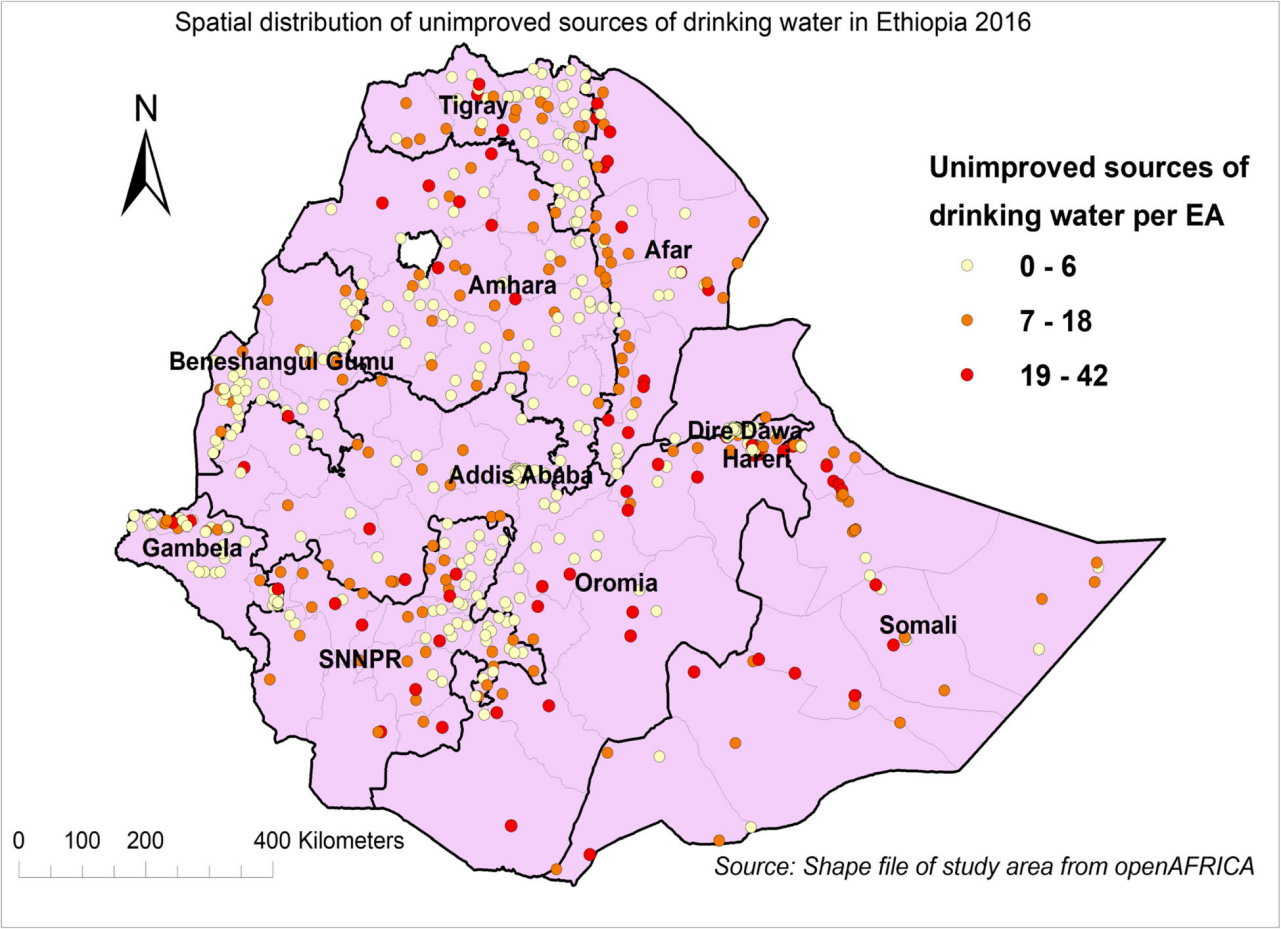
Spatial distribution of unimproved sources of drinking water in Ethiopia, EDHS 2016. Each spot (point data) on the plot represents one EA which encompasses the number of unimproved sources of drinking water (cases). The red color spots indicate areas with high rates of cases

The spatial distribution of unimproved sources of drinking water in Ethiopia varied from region to region. As shown in the Fig. 1, there were variations in the number of sources per enumeration area. The number of unimproved sources of drinking water per enumeration area was greater in Amhara region (particularly south and north Wollo, south Gondar, Waghemra, and East Gojam zones); the border areas of Tigray and Afar regions (eastern and southern Tigray and zone 2 of Afar); Southern nation and nationalities and peoples (SNNP) region (Keffa, Gamo Gofa, Konta, Dawro, and Bench Maji zones); Oromia region (southern part of Jimma zone); and Somali region (Fafan and Jarar areas) (Fig. 1).

**Fig. 2:**
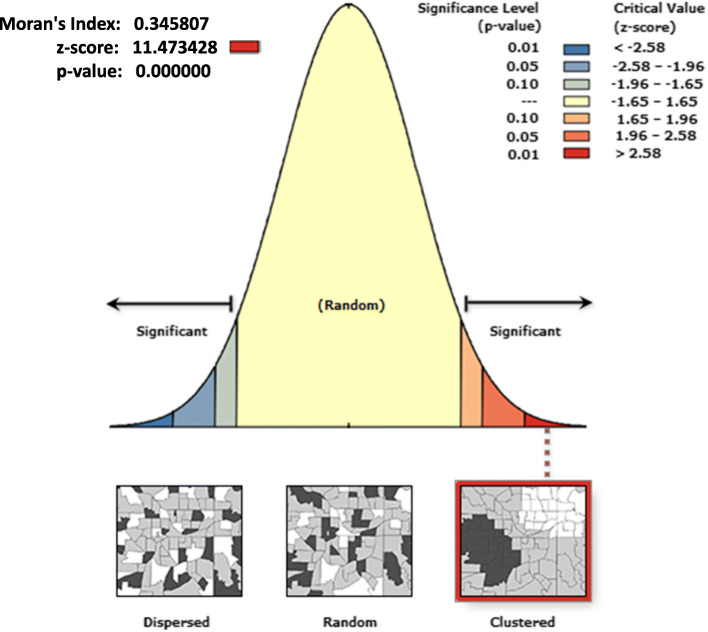
Spatial patterns of unimproved sources of drinking water in Ethiopia, EDHS 2016. The cluster pattern on the right side shows high rates of cases occurred over the study area. The output has automatically generated keys on the right and left top sides of the plot. An auto-generated interpretation shows that the likelihood of clustered pattern occurred by random chance is less than 1 %. The bright red and blue colors to the end tails of the plot indicates increased significance level

The spatial pattern of unimproved sources of drinking water was non-random all over the study area. The Global Moran’s *I* index was 0.35 which indicated that there was significant clustering of unimproved sources of drinking water in the country (Fig. 2).

### Spatial scan statistical analysis of unimproved sources of drinking water

Based on purely spatial analysis, scanning for clusters with high rates using the Bernoulli model, a total of 143 significant clusters were identified. Of which, eight were most likely (primary) clusters and the rest 135 were secondary clusters. The first spatial window which contains primary clusters was located in Amhara (eastern boarder of the north shewa zone) and the Afar (zone 3 and 4) regions. It was centered at 9.798697 N, 40.380059E with 55.24 km radius, with a relative risk (RR) of 2.23 and Log-Likelihood ratio (LLR) of 78.89, at *p* < 0.001. Peoples living within the spatial window were 2.23 times higher risk of getting their drinking water from unimproved sources as compared to peoples living outside the spatial window (Additional file 1, Fig. 3).

**Fig. 3:**
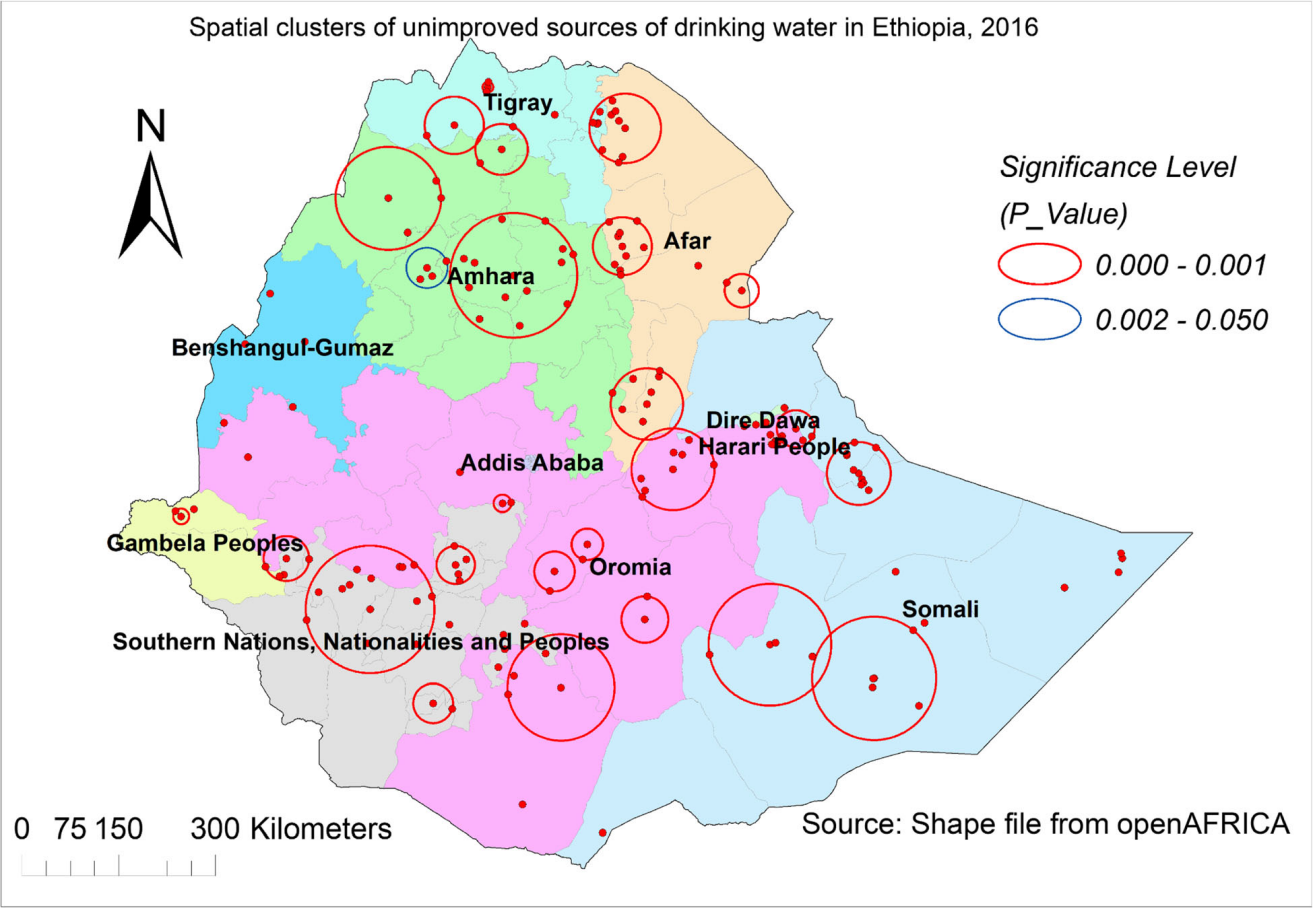
Most likely and secondary clusters of unimproved sources of drinking water in Ethiopia, EDHS 2016. The bright red and blue color rings indicate the most statistically significant spatial windows which contain primary and secondary clusters of unimproved sources of drinking water. Interpretation: Household members within the spatial window are at higher risk of getting their drinking water from unimproved sources than families outside the spatial window. The different color shadings on the map represented administrative regions (survey regions) of the country

The other 33 spatial windows which contain secondary clusters were found in different regions. Of which, two of them found in Amhara region (north and south Gondar, and north and south Wollo zones); three in Somali region (Shebele, Afder, Jarar and Fafan); one in Hareri region; three in Afar region (zones of 1, 2, 4); three in Tigray region (north western, western and eastern zones); two in western and eastern border of Dire-Dawa; more than 9 in Oromia region (east and west Haregre, Bale, Arsi, Guji, south west Showa, Jimma, Kemashi, Kelem Welega zones); more than 5 in SNNP region (Keffa, Dawro, Konta, Hadya, Sheka, Segen peoples); and two in Gambela region (Nuer and Majang). An additional file shows this in more detail (Additional file 1, Fig. 3).

## Discussion

This study shows that unimproved sources of drinking water at the national and regional level are non-random. Significant hotspot areas of unimproved sources of drinking water were identified in eight regions and one city administration. In a 2016 survey, the spatial scan statistics detected statistically significant clusters which could help programmers and policy makers to make appropriate decisions at the regional level. Though there is a lack of evidences as comparisons of this kind of study at local and national levels, this discussion was justified more of as per professional views. Of course, this finding, in agreement with another study, shows that the majority of rural households continued to rely on unimproved water sources [22].

In the first spatial window of primary clusters, 85% of households used their drinking water from unimproved sources. The hotspot areas were found in the junction area of the two regions (eastern Amhara and western Afar). It may be due to lack of attention given to peripheral and rural areas of the regions [11]. In another perspective, the government and partners may not give priority to populations who have better access to water sources without considering its quality. This idea is supported by a national survey, which stated that availability and sufficiency of drinking water, regardless of quality, is higher for unimproved sources of drinking water, and thus for rural areas [5].

The 33 spatial windows that contain secondary clusters in different regions were found almost in peripheral areas of their respective regions and city administration. It is known that access to education is low in the border areas as compared to the central areas. If so, the less educated people may be less informed about how to protect their available sources, and change to improved sources of drinking water. This is in line with a study done in sub-Saharan Africa [23].

The two spatial windows show hotspot areas of northern and northeast Amhara region. Like many other rural areas of sub-Sahara African countries, maintaining water source facilities and managing their operations in a sustainable way are still extremely challenging in rural areas of the Amhara region. Most water sources (facilities) in the region are under threat of losing functionality if the practice of operation and maintenance is not improved and changed to unimproved sources [24]. In another perspective, community based poor perception about improving water sources might be the cause of the hotspots which was agreed to a study conducted in the region [25].

The three spatial windows including hotspot areas of Somali region may show that shortage of water in Somali region has devastated due to the ongoing multi-year drought. While climate resilient water source development is a key to mitigate negative impacts of the drought. The majority of the population in Somali region is still dependent on seasonal water harvesting ponds, which are categorized as unimproved sources of drinking water (UNICEF Ethiopia, 2017, The Government of Sweden grants US$ 2.5 million to UNICEF for emergency response, unpublished). This phenomenon is similar to other regions like Afar; Gambela; boarder areas of SNNP, Oromia, Harari and Tigray regions; and rural and peripheral parts of Dire Dawa city administration [26]. Consequently, children living in hotspot areas are highly exposed to diarrheal diseases [27]. However, further small area investigation will be recommended to find the exact placement of unimproved sources of drinking water.

Since this study is based on nationally representative data, its findings may have implications for the national water, sanitation and hygiene (WASH) policy as well as the respective sectors’ and partners’ programs. Better resources, shall be allocated and mobilized for these hotspot areas. Mobilizing the users to protect their drinking water sources (unimproved) should be encouraged with locally-owned materials and skills. It is believed that only government and / or partner efforts are not enough to combat the problem. So, it is important if community-based awareness (aiming to water source protection) is created through students’ initiatives after schools’ curriculum revisions. This result also suggests the researchers to conduct further studies focusing spatial disparities of UISDW on the urban-rural, socioeconomic status, and climatic condition of households.

### Limitations of the study

In order to ensure that respondent confidentiality is maintained, The DHS program randomly displaced the GPS latitude/longitude positions (up to 2kms for urban and up to 5kms for rural clusters) for all DHS, Malaria and AIDS Indicators Surveys. Consequently, this study does not show the exact location of unimproved sources of drinking water in the study area. However, since the displaced cluster’s coordinates are located within the same admin0 (national), admin1 (survey region), and admin2 (zone) areas as the un-displaced cluster, it will not affect this spatial analysis result. Additionally, this analysis did consider neither in differences of urban-rural nor socioeconomic status of households. Since this study depends on main drinking-water sources, it will not reflect multiple water sources that used for multiple purposes.

## Conclusion

This study identified non-random clusters and hotspots of unimproved sources of drinking water in different regions of the country. Though improvement was shown at national level, the proportion of households using unimproved water sources are still high in the hotspot areas and still far apart from SDG and the national goal. Therefore, priority attentions may be needed in hotspot areas for resource allocation and control of related diseases.

## Supplementary information

**Additional file 1.** Significant spatial windows which contain primary and secondary clusters of unimproved sources of drinking water in Ethiopia, EDHS 2016. A cluster is statistically significant when its LLR is greater than the critical value, which is, for a significance level (Standard Monte Carlo Critical Values: 0.001: 11.90; 0.01: 10.32; 0.05: 8.72).

## Data Availability

The data to produce this manuscript are available and the author is prepared to share the customized dataset on request recognizing the benefits of such transparency. Otherwise, the original dataset can be accessed through www.dhsprogram.com after subscription and being an authorized user.

## Abbreviations

DHS: Demographic and health survey
EA: enumeration areas
EDHS: Ethiopia demographic and health survey
LRR: Log-likelihood ratio
RR: Relative risk
SDG: Sustainable Development Goal
SNNP: Southern nation and nationalities and peoples
UISDW: Unimproved sources of drinking water
WHO: World Health Organization
